# MEG/EEG Group Analysis With Brainstorm

**DOI:** 10.3389/fnins.2019.00076

**Published:** 2019-02-08

**Authors:** François Tadel, Elizabeth Bock, Guiomar Niso, John C. Mosher, Martin Cousineau, Dimitrios Pantazis, Richard M. Leahy, Sylvain Baillet

**Affiliations:** ^1^McConnell Brain Imaging Centre, Montreal Neurological Institute, McGill University, Montreal, QC, Canada; ^2^INSERM U1216 Grenoble Institut des Neurosciences (GIN), Grenoble, France; ^3^Grenoble Institut des Neurosciences, Université Grenoble Alpes, Grenoble, France; ^4^Center for Biomedical Technology, Universidad Politécnica de Madrid, Madrid, Spain; ^5^Biomedical Image Technologies, Universidad Politécnica de Madrid and CIBER-BBN, Madrid, Spain; ^6^Epilepsy Center, Neurological Institute, Cleveland Clinic, Cleveland, OH, United States; ^7^McGovern Institute for Brain Research, Massachusetts Institute of Technology, Cambridge, MA, United States; ^8^Signal and Image Processing Institute, University of Southern California, Los Angeles, CA, United States

**Keywords:** magnetoencephalography (MEG), electroencephalography (EEG), brain imaging data structure (BIDS), open data, group analysis, good practice, reproducibility, open source

## Abstract

Brainstorm is a free, open-source Matlab and Java application for multimodal electrophysiology data analytics and source imaging [primarily MEG, EEG and depth recordings, and integration with MRI and functional near infrared spectroscopy (fNIRS)]. We also provide a free, platform-independent executable version to users without a commercial Matlab license. Brainstorm has a rich and intuitive graphical user interface, which facilitates learning and augments productivity for a wider range of neuroscience users with little or no knowledge of scientific coding and scripting. Yet, it can also be used as a powerful scripting tool for reproducible and shareable batch processing of (large) data volumes. This article describes these Brainstorm interactive and scripted features via illustration through the complete analysis of group data from 16 participants in a MEG vision study.

## Introduction

Magnetoencephalography (MEG)/EEG data analysis requires translating neuropsychology and cognitive neuroscience questions into electrophysiology hypotheses. This in turn requires the design of an analytical workflow for signal extraction and analysis, possibly involving source imaging and model estimation (Baillet, 2017). With Brainstorm, we deploy tools and solutions for this purpose for use by a broad cross-section of neuroscience researchers (Tadel et al., 2011). We provide extensive online documentation and user support, with large collections of web tutorials for MEG, EEG, depth recordings and various types of study design. Brainstorm also embodies the philosophy that even sophisticated data analysis in electrophysiology benefits from a level of interactive visual assessment of data quality and of the spatio-temporal characteristics of possible effects within/between experimental conditions. Therefore, Brainstorm produces a variety of graphical and quantitative reports, for both point-and-click user interactions and automated data processing pipelines. All together, we believe these elements make Brainstorm a comprehensive yet accessible application for sophisticated and reproducible neuroscience research. Here we emphasize these unique software features in the context of a typical group data analysis workflow.

We used simultaneous MEG/EEG recordings from 16 participants performing a simple visual recognition task from presentations of familiar (from celebrities), unfamiliar and scrambled faces. The original data was published by Wakeman and Henson (2015) and is also featured in the SPM tutorial “Multimodal, multisubject data fusion”^1^. We used the version of this dataset organized according to the new MEG-Brain Imaging Data Structure (BIDS^2^) (Gorgolewski et al., 2016; Niso et al., 2018).

We feature two specific aspects of data analysis with Brainstorm: We first describe the interactive processing for one typical subject, from preprocessing of raw data to the extraction of event-related responses, the production of time-frequency decompositions and source modeling. We then describe how to transfer this analysis to the full group of 16 participants and derive group-level inferential statistics. Reproducing the analyses presented here can be done by following the new online tutorials created as online complements to this paper^3^. Users new to Brainstorm will also benefit from our comprehensive introductory tutorials^4^.

## Data

The original study concerned the identification of brain responses specific to faces and their familiarity to participants. Subjects were presented series of still images from three categories: familiar faces, unfamiliar faces, phase-scrambled faces. Familiar faces were from celebrities known to all participants. They were asked to rate a feature of no interest after each stimulus presentation, namely the left–right symmetry of the presented image. Six 10-min acquisition runs were collected from each participant, for a total of about 300 trials per stimulus category. As our goal here is to demonstrate software practicalities, we report solely on the early visual brain response within the first 300 ms post-stimulus, with an emphasis on the specific aspects of responses to faces vs. scrambled images (familiarity was not a factor of interest in the present analysis); see (Wakeman and Henson, 2015) for a complete report on all aspects of the study.

The data were recorded in 16 healthy participants with an Elekta Neuromag VectorView MEG system (102 magnetometers, 204 planar gradiometers), simultaneously with 70 scalp EEG electrodes with nose reference. The sampling rate was 1,100 Hz. Three fiducial points and the scalp surface were 3-D digitized for registration with M/EEG channel locations and structural MRI.

To replicate the results presented in the original article, we imported the version of the data corresponding to recordings processed with Elekta’s MaxFilter: they are available from the “derivatives” folder of the MEG-BIDS distribution of the data. MaxFilter was used to attenuate environmental noise with signal space separation (SSS), detect bad channels, apply a notch filter to reduce powerline artifacts, compensate for head movements and align the data across runs to match the head position at the start of the fourth run; see (Wakeman and Henson, 2015) for details. For noise modeling purposes, we used empty-room MEG noise recordings acquired between 0 and 6 days from acquisition of subject data: they were also processed with MaxFilter, in an identical manner as the participants MEG data.

Structural MRI data was acquired on a 3T Siemens TIM Trio (1 mm × 1 mm × 1 mm, T1-weighted). The MR data volumes were de-faced for further subject de-identification. The MEG-BIDS data repository includes the anatomical segmentation produced by FreeSurfer 5.3^5^ (Dale et al., 1999).

## Download and Installation of Data and Software

All the files featured in this communication are available from openneuro.org, https://openneuro.org/datasets/ds000117. The full analysis requires a total of 400Gb of available disk space. To reproduce the analyses presented here readers should download and install Brainstorm^6^.

The interactive environment of Brainstorm can be run without a Matlab license. However, users without a Matlab license cannot execute Matlab scripts and therefore cannot run the automated scripts that reproduce the analyses presented here. Brainstorm scripting requires Matlab 2008a or later.

## Single-Subject, Single-Run Data Analysis

We first present analyses performed on one data run from one of the participants. We then show how the resulting pipeline can be extended to other runs and other participants, prior to group statistical inference.

At the core of Brainstorm’s architecture and user experience is a data manager tool, which facilitates data organization and sorting by experimental conditions and groups of participants. Brainstorm exploits the principled data organization of MEG-BIDS: all data volumes (raw and FreeSurfer processed MRI, MEG/EEG) can be readily registered at once to the Brainstorm database following the menu “File > Load protocol > Import BIDS dataset”^7^. However, here we describe the steps necessary to import multimodal data into Brainstorm so that the analysis described below can be extended to new data sets. If data are not organized using the MEG-BIDS structure, these operations need to be repeated for all runs and participants.

### Structural MRI: T1 Volume and Derivatives

**FIGURE 1:**
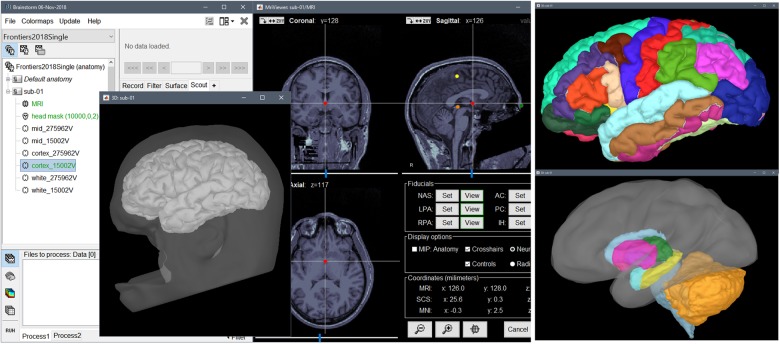
Brainstorm screenshot after importing anatomical MRI data and FreeSurfer derivatives. From **(left)** to **(right)**: Brainstorm database explorer, 3-D rendering of head and pial surface meshes (the scalp surface and MRI volume were de-faced for anonymization purposes), orthogonal views of T1 MRI volume and anatomical fiducials, FreeSurfer/Mindboggle cortical parcellation, FreeSurfer/ASEG volume atlas of subcortical and cerebellar structures.

We created a new study or *protocol* in the software database named “Frontiers2018Single,” to which we added a new subject with “sub-01” as coded ID. By right-clicking on the *subject* folder and selecting “Import anatomy folder” in the contextual menu, we loaded all the MRI data from the FreeSurfer folder of the downloaded dataset “derivatives/freesurfer/sub-01/ses-mri/anat.” These included the individual structural MRI, the tessellated cortical surfaces and anatomical atlases registered to the individual anatomy^8^. The scalp surface was reconstructed automatically by Brainstorm from the T1 volume data. Figure 1 shows a screen capture of typical Brainstorm structural (surface and volume data) MRI displays.

Brainstorm computes a 4 × 4 affine transformation that registers the subject’s T1 MRI to the MNI coordinate system using the *spm_maff8* function (included in Brainstorm’s distribution) from SPM12 (Ashburner and Friston, 2005). Brainstorm also sets default coordinates for anatomical fiducials (NAS = nasion, LPA = left ear, RPA = right ear) for registration with MEG coordinates. These approximate points are based on standard MNI coordinates. The actual individual locations of these reference points are also available from the MEG data file and are used to initialize MEG/MRI coregistration. In principle, as few as three points are sufficient for registration but more robust alignment can be readily achieved using the individual digitized head shape (see next section), as with the present dataset^9^. When the individual head shape is not available or has poor quality, the positions of the NAS/LPA/RPA fiducials must be defined manually using Brainstorm’s MRI Viewer.

Alternatives to FreeSurfer can be used to import MR derivative data into Brainstorm: BrainVISA (Rivière et al., 2003), BrainSuite (Shattuck and Leahy, 2002) and CIVET (Ad-Dab’bagh et al., 2006). All of the above generate cortical surface meshes, yet only FreeSurfer and BrainSuite readily provide registration of individual data to atlases, which eventually facilitates the projection of MEG/EEG individual source maps to a common anatomical template across the group.

### MEG/EEG and Registration With Structural MRI

#### Raw MEG/EEG Files

The continuous FIF file from the first MEG acquisition run of sub-01^10^ was added to the Brainstorm database using the “Review raw file” contextual menu over the subject entry. This operation creates a link to the original raw data file, whose contents can be reviewed and manipulated without requiring Brainstorm to duplicate the raw data file^11^. The product of most of the following pre-processing steps is efficiently saved in this link file without changing the contents of the original raw data file.

We ignored two options offered interactively while linking the recordings to the database: automatic registration with MRI and importation of stimulus triggers. We describe below how to proceed with these issues manually.

**FIGURE 2:**
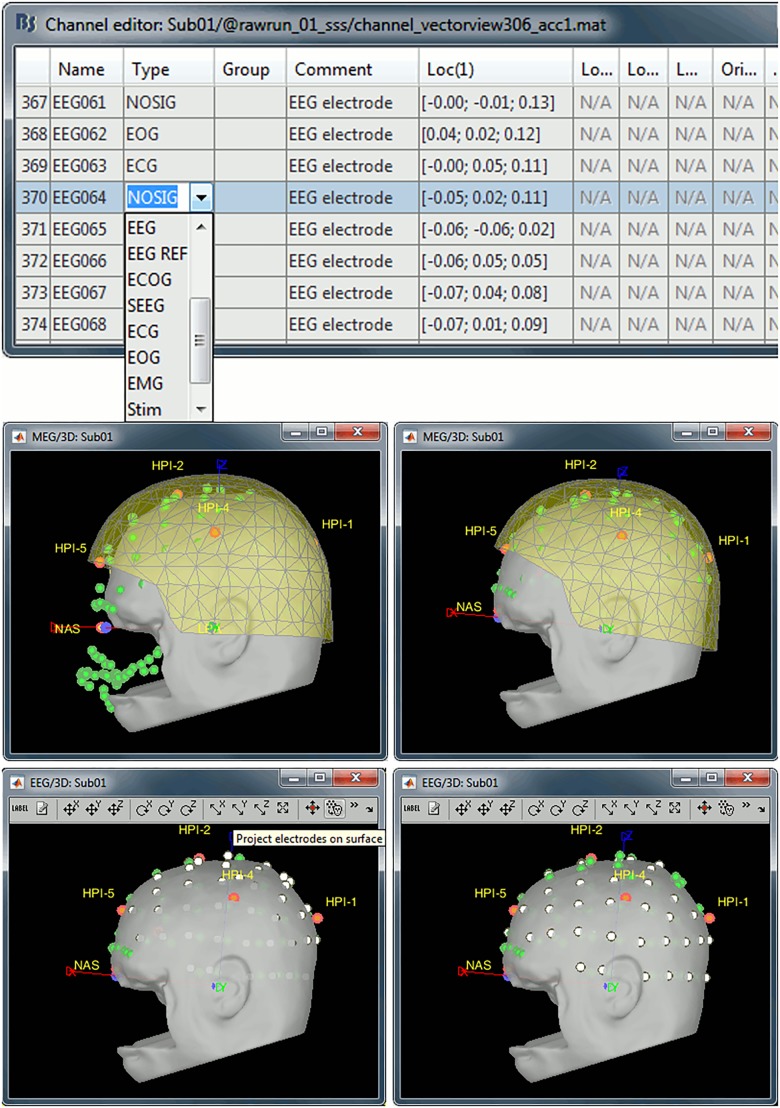
**(Top row)** Manual editing of channel types, removal of digitized head points below the nasion, and fit of remaining points to the scalp surface; **(bottom row)** projection of EEG electrodes onto scalp surface. Green dots: digitized head points, white dots: EEG electrodes, gray surface: scalp surface extracted from MRI, yellow surface: inner surface of MEG helmet.

A channel file is created next to the link to the raw recordings: this database entry describes the data channels available (names, types, 3D positions). For clarification, we manually edited the types of some channels, using Brainstorm’s channel editor: EEG062 was changed to EOG (electro-oculogram), EEG063 to ECG (electrocardiogram), EEG061 and EEG064 to NOSIG (no signal) (Figure 2).

#### Registration With Structural MRI

The registration of MEG sensors with structural MRI data was initialized with the alignment of the NAS/LPA/RPA fiducial points as identified from the MRI MNI coordinates and their digitized locations in the MEG file. Brainstorm can refine this geometrical alignment by estimating an optimal rigid-body transformation that fits multiple digitized head points (expressed in the same referential frame as the MEG sensors) to the scalp surface (automatically extracted from T1 MRI by Brainstorm). Note that the MRI data was de-faced, so head points below the nasion were discarded by selecting the popup menu item “Digitized head points > Remove points below nasion.” We then used the menu item “MRI registration > Refine using head points” to fit the digitized scalp points, augmented with the EEG electrode locations, to the head surface, using an iterative closest point algorithm (ICP).

One last step consisted of projecting the EEG electrodes onto the scalp, thereby removing any distance between their 3-D digitized locations and the subject’s actual scalp surface. This step is important for accuracy of the EEG forward model. This projection is obtained via the menu item “MRI registration > EEG: Edit,” followed by “Project electrodes on surface.”

We recommend visual inspection of the registration outcome for MEG and EEG sensors, especially before subsequent source estimation, as incorrect coregistration negatively affects the accuracy of source modeling (Figure 2).

#### Definition of Experimental Events

A stimulus trigger signal marked the presentation times of stimulus images on the screen, with different codes for the three experimental categories. These signals were recorded on channel STI101 in the present data. There was a known, constant delay of 34.5 ms between each trigger pulse in STI101 and the actual presentation of the stimulus image. The following binary event codes were used at the time of data acquisition (bit number 3 coded for face, bit 4 for unfamiliarity, and bit 5 for the scrambled stimulus): Familiar faces: 5 (00101), 6 (00110), 7 (00111); Unfamiliar faces: 13 (01101), 14 (01110), 15 (01111); Scrambled images: 17 (10001), 18 (10010), 19 (10011). There are other event codes available in the recordings, but they are not described in the README file distributed with this dataset.

The read-out of these markers can be performed via the interactive menu item “File > Read events from channel” in the Record tab^12^. Here, we illustrate the features of Brainstorm’s pipeline editor to achieve the same purpose. Note that the resulting pipeline operation can be applied at once as a batch procedure on all runs and participants.

After dragging and dropping the link to the continuous file in the Process1 panel at the bottom of the main Brainstorm window, we clicked on the Run button to open the pipeline editor. We then selected “Events > Read from channel” with the option “Bit: detect the changes for each bit independently” from the list of available process operations. This created one event for each bit of the integer value recorded on channel STI101.

We considered three stimulus categories with values: bit 3 = face, bit 4 = unfamiliar, bit 5 = scrambled. We created the categories “Unfamiliar” (bit 3 = 1, bit 4 = 1, bit 5 = 0), “Familiar” (bit 3 = 1, bit 4 = 0, bit 5 = 0), and “Scrambled” (bit 5 = 1). We discarded all other event categories originally in the data, for clarity. This step was performed via Brainstorm’s pipeline editor, by combining the processes “Events > Group by name,” “Rename event,” and “Delete events,” in that order.

**FIGURE 3:**
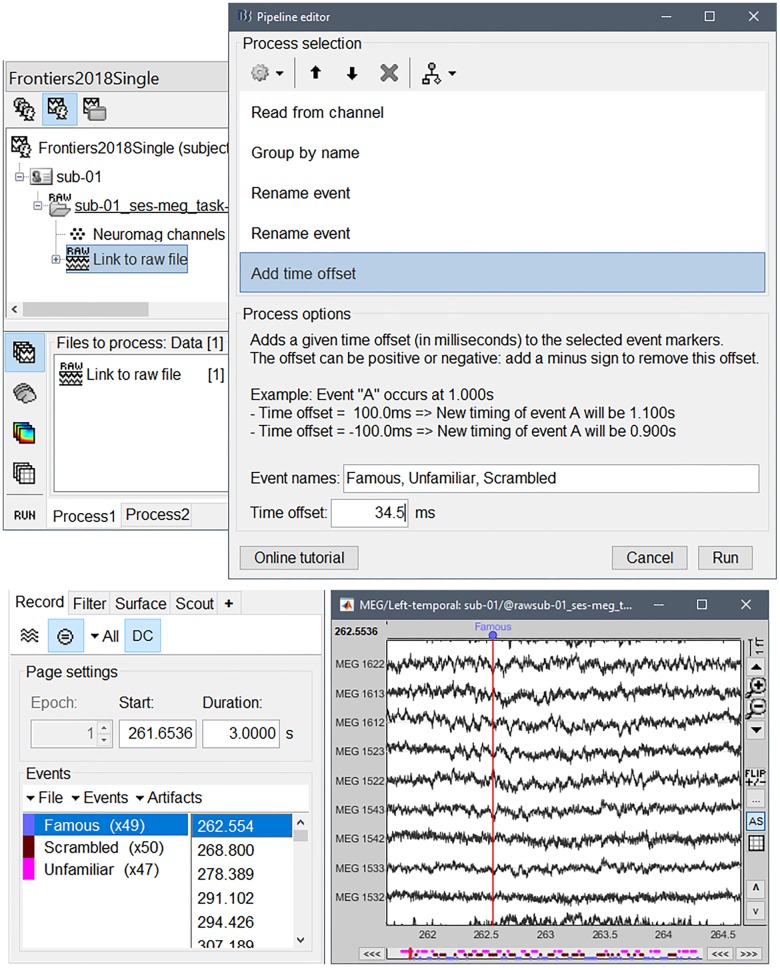
**(Top)** Selection of files in Process1 and pipeline editor; **(bottom)** reviewing the data traces around the event markers created for the three categories of visual stimuli (familiar, unfamiliar, and scrambled).

Finally, we adjusted the timing of all detected events to compensate for the 34.5-ms presentation delay, with process “Events > Add time offset” (Figure 3).

### Pre-processing and Data Review

#### Power Spectrum Estimation

We recommend estimating and reviewing power spectra of MEG/EEG sensor traces for basic quality control. Bad channels, episodes of major signal alterations, artifacts (breathing, dental work, muscle, and eye movements) and environmental noise (stimulation devices, power lines, head localization coils) can readily be identified by a trained user^13^.

**FIGURE 4:**
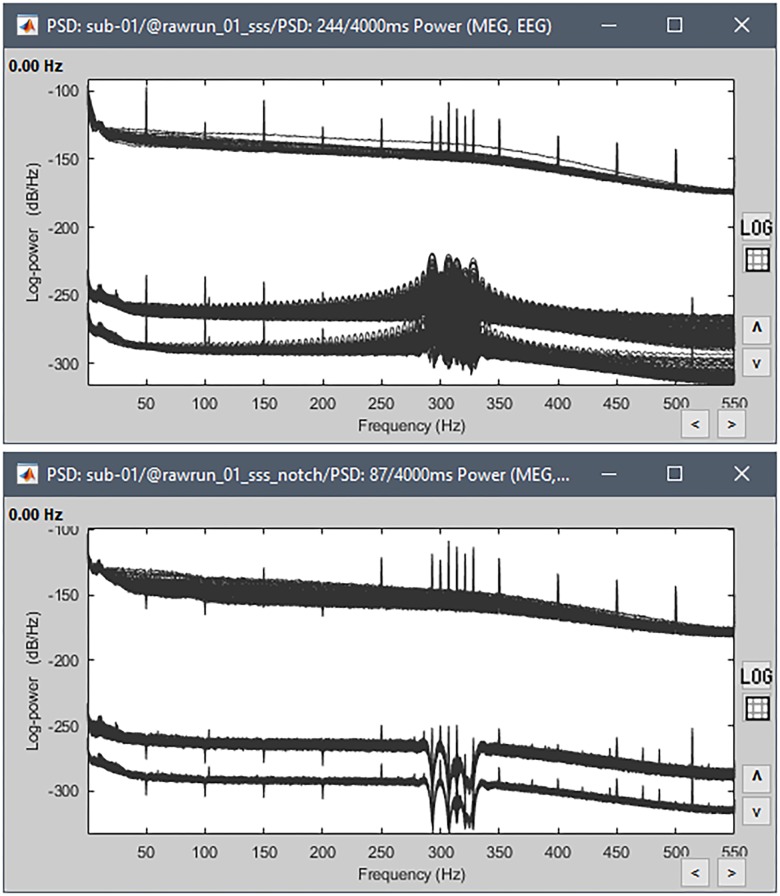
Power spectrum density, log-scaled. **(Top)** Estimated from the full recordings; **(Bottom)** after excluding the cHPI transition, applying the notch filters, excluding bad channel EEG016 and re-referencing the EEG. Each type of sensor has a different range of amplitude, creating three blocks of channels in these Figures – from top to bottom: EEG, MEG gradiometers, MEG magnetometers.

To obtain an estimate of the power spectrum density (PSD) on all channels, we dropped the raw-data link in Process1 and selected process “Frequency > Power spectrum density (Welch)” (window duration 3 s with 50% overlap). The PSD estimates were saved in a separate file attached to the original data and can be readily reviewed for all channel types (Figure 4).

We noted the presence of large side lobes around about 310 Hz in the spectrum of all MEG channels, which points to signal discontinuities in the recordings. Indeed, while continuous head tracking was used in this acquisition, it was only activated at the time of the stimulation. The file starts at time 226 s, with inactive Head Position Indicator coils (HPI). At 248 s, the HPI coils were turned on, yielding an abrupt, step transition of high-amplitude sinewave signals. Shortly after, MaxFilter started filtering out the HPI signals and correcting for head movements, yielding a second step in the MEG recordings. Such abrupt transitions create large distortions of the power spectra. We therefore marked the first 22 s as a bad data segment with the process “Events > Detect cHPI activity (Elekta)” and recomputed the power spectra from clean portions of the recordings only.

Figure 4 (lower) shows the power spectra of this truncated data. Physiological peaks can be observed at 10 Hz (alpha rhythm), 50 Hz and harmonics (powerline contamination in United Kingdom) and peaks at 293, 307, 314, 321, and 328 Hz (from Elekta electronics, including residuals of HPI signaling) and an unknown source at 103.4 Hz. We also noticed that channel EEG016 was noisy, with a power spectrum oddly standing above the other EEG channels’. We therefore inspected the traces from this electrode when reviewing the recordings (see below) and the channel was ultimately excluded from further analysis.

#### Frequency Filters

Power line contamination can easily be removed with notch filters centered at 50 Hz and harmonics (100 Hz, 150 Hz, and higher). We restricted our analyses to that of event-related stimulus responses below 32 Hz, the frequency range analyzed by Wakeman and Henson (2015). Since this is below 50 Hz, notch filtering is not required. In addition, powerline contamination is greatly reduced by stim-locked averaging because of the random phase of the interference from trial to trial. We did perform notch-filtering for the sake of generalizability of the pipeline presented here. Note also that more advanced data analysis based on single-trial measures, including that of specific and/or faster oscillatory components, may require more thorough artifact correction.

Frequency filters need to be applied depending on data quality and the signal components of interest to meet the scientific aims of a given study. High-pass filters (HPFs) remove the arbitrary DC offset and slow baseline drifts of MEG sensors (<0.2 Hz), physiological artifacts due to breathing and slow eye movements. The cutoff frequency must be selected carefully, especially when relevant slow brain responses are expected, e.g., in working memory retention periods. Low-pass filters (LPFs) remove high-frequency contaminants such as muscle artifacts and physiological stimulators. LPFs also restrict the useful frequency range that can be analyzed.

It is important to be aware that filters can generate transient effects at the beginning and end of each signal trace. The length of this transient depends on multiple factors, with narrow-band filters and very low frequency cut-off HPFs inducing longer edge effects. For this reason, it is preferable to apply frequency filters directly on continuous signal traces, before shorter epochs of interest are extracted^14^.

Here we applied notch filters on the continuous recording by dropping its file link into the Process1 panel and ran “Pre-process > Notch filter” at 50, 100, 150, and 200 Hz. Brainstorm applies 4th order IIR notch filters with zero-phase lag. The process creates a filtered version of the continuous file with suffix “_notch” saved in Brainstorm format. Other filter types (band-pass, low-pass, high-pass, and band-stop) can be applied following the same procedure.

#### Bad Channel Identification

**FIGURE 5:**
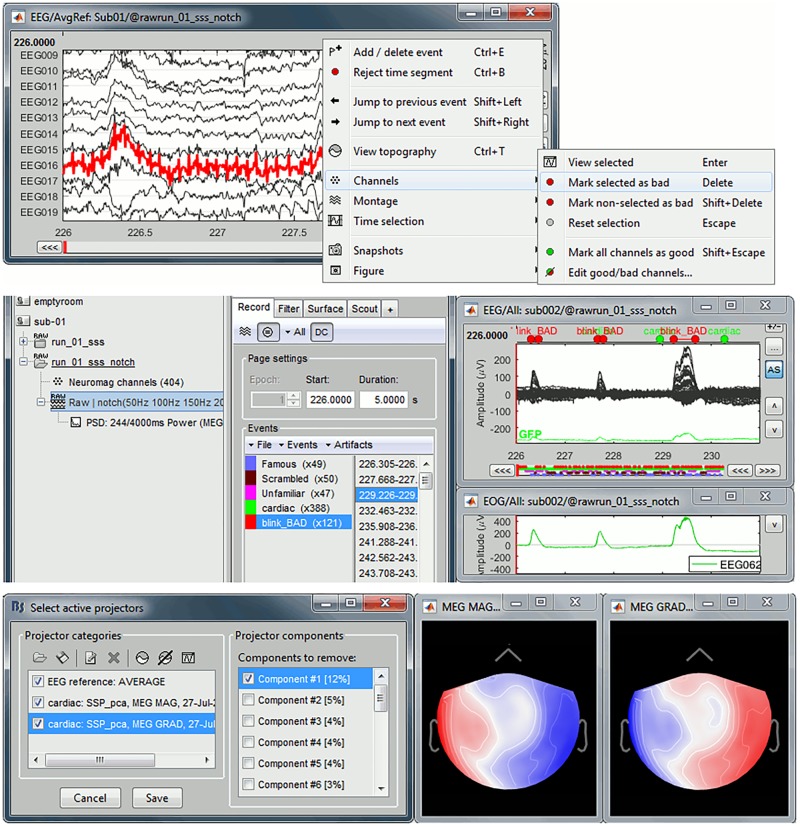
Data cleaning. **(Top)** Bad channels are visualized, marked as red traces and discarded in further processing steps. **(Middle)** Eye blink detection in EOG traces (green), with corresponding artifact in MEG traces (black). **(Bottom)** SSP components corresponding to heartbeat events and corresponding magnetometers (left) and gradiometers (right) surface topography.

The PSD plots revealed that EEG016 had poor signal quality. Bad-channel labeling can be performed interactively^15^: we displayed the EEG traces by right-clicking on the filtered file and selected the EEG/Display time series contextual menu item. We then selected channel EEG016 from the display and marked it as bad after another right click (Figure 5). Note that keyboard shortcuts are available for this and many other procedures in Brainstorm.

Bad channels need to be marked before running further cleaning procedures that combine values from multiple channels, such as those described in the sections below.

#### EEG Referencing

After removing EEG016 and for visualization purposes, we re-referenced the EEG traces with respect to the instantaneous average across all remaining EEG signals. This was done via the montage menu in Brainstorm by selecting the “Average reference” entry^16^. Note that montages are for display only: they do not alter the signals stored in the file. Permanent alteration of EEG referencing is via process “Standardize > Re-reference EEG,” which simply applies a linear re-referencing operator to the original data, for data storage efficiency. The list of linear operators applied to a file can be retrieved selecting the menu item in the file viewer “Artifacts > Select active projectors” from the “Record” panel. The original EEG referencing can be recovered by simply deleting the “EEG reference: AVERAGE” entry from the list displayed.

#### Removal of Eye Blinks

Brainstorm features solutions for detecting eye blinks via the process “Events > Detect eye blinks,” which creates event markers at local maxima of EOG traces caused by eye blinks. The MEG/EEG artifacts can then be removed by designing specific SSP signal projectors from the signal statistics of blink events. In the present study, the focus was on brain responses related to visual stimulus presentations. Therefore, we opted to exclude rather than correct the epochs where blinks were detected.

We opened the EOG traces by right-clicking on the MEG/EEG file and selected the contextual menu item “EOG > Display time series.” We reviewed the signal traces from EEG062 to define the amplitude threshold for the detection of blinks. We chose 100 μV for sub-01 and ran process “Events > Detect events above threshold” on EEG062, in the frequency band 0.3–20 Hz, to automatically mark blink data segment labeled “blink_bad.” Note that with Brainstorm, all event with label names that include the tag “bad” are automatically excluded from further analysis (Figure 5).

#### Correction of Heartbeat Artifacts

Heartbeats are another common source of artifacts in MEG and EEG recordings. Although their contribution to event-related average signals can be small, their removal is considered a good practice (Gross et al., 2013). We selected the filtered continuous file from Brainstorm’s data manager and ran the process “Events > Detect heartbeats” on ECG channel EEG063. The built-in detection algorithm identified R-peaks in the electrocardiogram trace, which are synchronized to the MEG artifacts^17^.

We derived signal space projectors from the signal statistics about heartbeat events (Uusitalo and Ilmoniemi, 1997). The technique is based on the principal component analysis (PCA) of MEG traces contaminated by heartbeat sources, and orthogonal projections away from the corresponding spatial patterns. We opted for SSP instead of independent component analysis (ICA, also available in Brainstorm, Makeig et al., 1996), because it is faster and more specific of the source of artifacts^18^.

We ran the Brainstorm process “Artifacts > SSP: Heartbeats” first on MEG magnetometers (“MEG MAG” channel type), then on MEG gradiometers (“MEG GRAD”). The orthogonal projectors produced can be reviewed and selected interactively (“Select active projectors”). Another benefit of SSP is that PCA components are ordered according to their contribution to signal variance. Brainstorm features a dedicated graphical user interface (GUI) to review and select the SSP components of contaminants: we selected “Display component topography” in that GUI (Figure 5), to review the effect of removing the first SSP component from MEG traces. We verified visually that by doing so, it efficiently removed heartbeat artifacts without obviously affecting signal traces away from ECG events. This is best verified by visualizing lateral MEG sensors and toggling the application of SSP projectors back and forth. We selected the first SSP component for the magnetometers and gradiometers decompositions for sub-01/run-01 for the further processing steps.

#### Marking of Additional Bad Segments

Segments of MEG/EEG traces can be spoiled by other sources of nuisance: Body and head movements, transient flux jumps from SQUID sensors, and uncontrolled environmental sources (building vibrations, elevators, cars, or trains, etc.)

We recommend that traces are systematically reviewed for visual detection of obvious episodes of signal contamination. Brainstorm features rapid browsing capacity of virtual pages of customizable duration (typically 20–30 s) and sensor selections. Further automatic processes help expedite such quality control: The process “Artifacts > Detect other artifacts” identifies time segments that contain typical artifacts from eye and head/body movements or muscle contractions in pre-determined frequency bands, where such signal contamination is the most commonly observed (1–7 Hz for body movements, eye movements and dental work; 40–240 Hz for muscle contractions, etc.; Gross et al., 2013). This process creates new event markers to expedite subsequent visual inspection and validation.

Another estimation of the sensor traces’ PSD after pre-processing confirms that all the cleaning steps worked as expected (Figure 4, bottom).

### Event-Related Epoching

We performed the temporal segmentation of continuous data about each event of interest (i.e., visual stimulus presentations) by selecting “Import in database” from the contextual menu over the filtered version of the continuous file^19^ (epoching is also available from the process “Import recordings > Import MEG/EEG: Events”). We selected the *Familiar, Unfamiliar*, and *Scrambled* event categories from the list displayed, and defined the epoch as [-500, 1200] ms about each event. This epoch duration is long enough to capture all event-related brain responses of interest and to define a pre-stimulus baseline for subsequent standardization procedures. It also provides additional temporal padding to absorb further filtering edge effects when applied to single trial data segments (as with low-pass filtering, time-frequency decomposition and connectivity analysis). Yet, it is also short enough not to overlap with brain responses of interest from previous or subsequent trials.

As we import these epochs, we also apply a correction for the arbitrary DC offset observed in the MEG sensors. In MEG, the sensors record variations around an arbitrary level, therefore this operation is always needed, unless it was already applied during one of the pre-processing steps (e.g., a high-pass filter can efficiently replace this DC correction). We corrected the DC offset of every sensor at each trial by selecting the option “Remove DC offset: Time range = [-500, -0.9] ms” at the time of epoching. This can also be achieved as a pipeline step, choosing the process “Pre-process > Remove DC offset” with the same baseline definition. We applied such baseline correction to MEG and EEG traces, as specified in the “Sensor types” field of the related processes.

**FIGURE 6:**
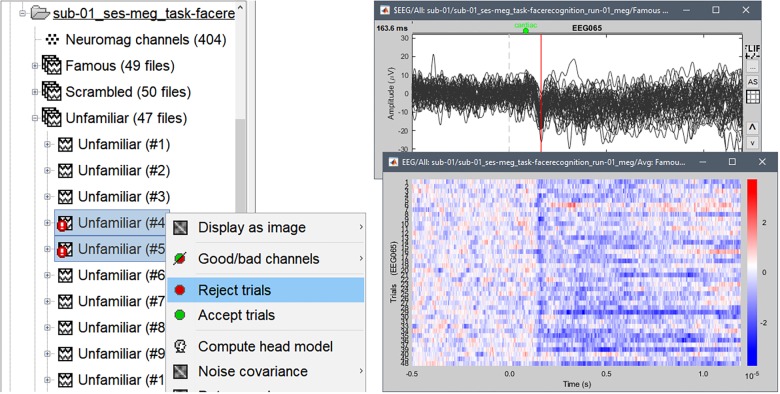
**(Left)** Accepting or rejecting single trials using the database explorer. **(Right)** Single-trial traces for electrode EEG065 superimposed as cluster **(top)** and raster **(bottom)** plots over 40 good trials from the Familiar condition, with a low-pass filter below 32 Hz applied for display purposes only.

The single trials for all 3 experimental conditions were imported in the database, each trial data being stored in a separate file. If a trial co-occurred with a “bad” event or segment, Brainstorm marked it as bad, which *de facto* excluded it from further analysis. We found in sub-01/run-01 that most bad trials were toward the end of the run, with more occurrences of eye blinks and body movements, likely due to subject fatigue. Note that trials can be toggled as good or bad via the database explorer, selecting the menu item “Reject trial” or “Accept trial” interactively (Figure 6). Single trials can be rapidly inspected visually, browsing through files using convenient keyboard shortcuts. Additionally, Brainstorm offers several visualization features for groups of trials, such as sensor-specific raster and cluster plots (Figure 6).

### Trial Averaging

We produced trial averages to obtain MEG event-related fields (ERFs) and EEG event-related potentials (ERPs) for each stimulus category for sub-01/run-01. Averaging across runs requires further attention as MEG sensor locations can vary between runs, because of head motion relative to the helmet. We discuss this aspect below in the context of group analyses.

**FIGURE 7:**
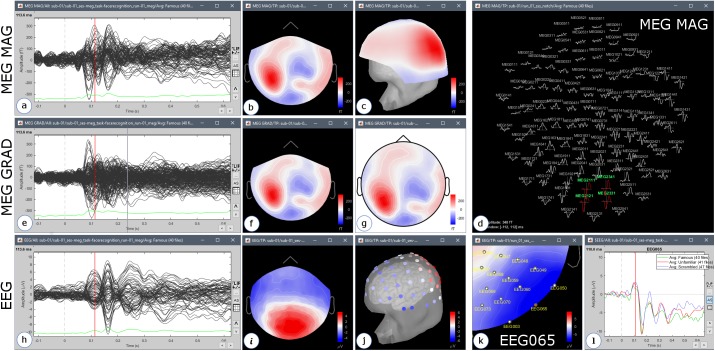
Examples of display of the average of 40 trials of the Familiar condition, 113 ms after the presentation of the visual stimulus, with an online low-pass filter at 32 Hz (subject 01, run 01) for display purposes only. The top row shows the recordings of the MEG magnetometers: **(a)** signal time series from 100 ms before to 600 ms after the stimulus; **(b)** 2D sensor topography at 113 ms; **(c)** 3D topography; **(d)** 2D Layout display showing the local shape of the signals around 113 ms. The middle row shows the MEG gradiometers: **(e)** time series, **(f)** 2D topography, **(g)** 2D topography projected on a disk. The bottom row shows the EEG recordings: **(h)** time series, **(i)** 2D topography, **(j)** EEG electrodes represented on the head surface with values recorded at 113 ms; **(k)** 2D topography zoomed around selected electrode EEG065, **(l)** values recorded by electrode EEG065 between –100 and 600 ms for the three conditions Familiar (green), Unfamiliar (red), and Scrambled (blue).

We selected all imported epochs and ran process “Average > Average files: By trial groups (folder average).” This created one event-related average data file per stimulus category (*Familiar, Unfamiliar*, and *Scrambled*). Brainstorm features a great variety of display options for event-related data: time series, several types of 2D/3D sensor topography plots, with interactive frequency filtering, etc. Several figures can be opened simultaneously, for different conditions and different modalities, and are synchronized: changing the time or sensor selection in one figure updates the other displays (Figure 7). The figure popup menu “Snapshot” lists several options to export Brainstorm figures to picture files and animations^20^.

### Source Modeling

We next obtained image models of the cerebral currents from the preprocessed MEG/EEG sensor time series. Brainstorm’s online tutorials describe in detail the forward and inverse modeling steps required^21^; see also (Baillet et al., 2001; Baillet, 2017) for reviews.

We emphasize that not all neuroscience questions require source modeling: differential sensor topographies and/or event-related component latencies, or more recent multidimensional classification techniques (Cichy et al., 2014) between experimental conditions may suffice to test neuroscientific hypotheses. But here we wish to verify whether the visual ventral pathway was more strongly activated in response to faces, especially in the fusiform area, within the first 200 ms after stimulus presentation (Schweinberger and Neumann, 2016).

We elected to use a distributed source imaging model rather than fitting an equivalent current dipole (ECD) model. The rationale was that we expected multiple brain regions to be activated simultaneously within the 0–200-ms time window of interest, which is challenging to the non-linear optimization problem of ECD dipole fitting (Baillet et al., 2001).

There are multiple options to distributed source modeling. We opted for constraining the positions and orientations of elemental current dipoles to the individual cortical surface of participants. We used 15,000 cortical elemental dipoles to cover the entire cortical surface – a number sufficiently large to sample the folded details of cortical anatomy. Brainstorm also features a range of simpler (equivalent moving dipole fits, unconstrained 3-D dipole grids in skull volume) and more sophisticated source models (including cortical and subcortical structures based on anatomical atlases of basal ganglia adjusted to individual anatomy^22^). In principle, the most complete anatomical model shall be preferred as the source space. However, adding more detailed structures also adds signal dimensions, increasing the ill-posedness of the inverse problem. It also increases the complexity and practical aspects of handling, storing, visualizing and interpreting the source models produced. For most studies, we find the cortically constrained model to be a reasonable tradeoff between completeness and complexity. Note that when the individual’s structural MRI data is not available, Brainstorm features anatomical templates that can be adjusted to the participant’s digitized scalp points or electrode locations^23^.

Magnetoencephalography and EEG forward models in Brainstorm include both Boundary Element Models (BEMs) based on individual tessellations of segmented head compartments^24^ and fast, analytical approximations of the head geometry with multiple nested spheres, where these spheres can also be locally fitted separately to each sensor (Leahy et al., 1998; Huang et al., 1999).

For the MEG forward model, we used the locally fitted sphere model while we used the BEM computed from the individual head compartments for the EEG forward model. In principle, because they represent true head shape, BEM models are superior to the spherical approximations. However, the latter can be more robust as they are not sensitive to issues that can limit BEM accuracy such as the effect of large triangle sizes in surface tessellations or proximity of a source to one of the mesh vertices. Generally, EEG source modeling is more sensitive than MEG to approximations of the head shape (Baillet, 2017) which in part motivated the different choices for MEG and EEG here.

**FIGURE 8:**
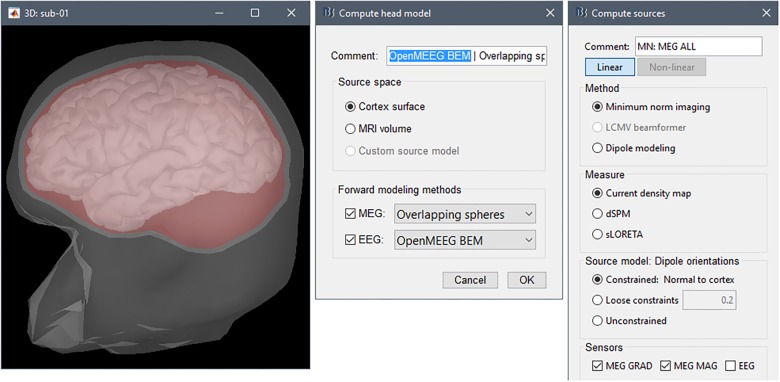
Source estimation. From **(left)** to **(right)**: BEM layers (scalp, outer skull, and inner skull) and cortex, forward model basic options, inverse model basic options.

Brainstorm’s BEM engine uses OpenMEEG (Gramfort et al., 2010). We produced the surface envelopes for scalp, inner skull and outer skull within Brainstorm, via a right-click selection of the “Generate BEM surfaces” contextual menu item over the Subject data folder. OpenMEEG developers recommend using dense meshes (e.g., 1922 vertices per layer), however, due to BEM memory requirements, we reduced the number of surface nodes of all tissue envelopes down to 1082 vertices for scalp, and 642 for outer and inner skull (Figure 8, center).

We then produced the actual EEG forward model by right-clicking on the channel file in the imported data folder and selecting the contextual menu item “Compute head model” (Figure 8, right). This generated the BEM model of the forward fields for 45,000 dipole triplets distributed on the cortex surface. Note that the final number of dipole sources in the model will be 15,000: Brainstorm’s forward modeling computes a generic forward field subspace at each of the 15,000 locations using triplets of orthogonal elementary dipoles (3 orientations × 15,000 vertices = 45,000); the orientation constraint is applied later at the moment of source modeling in the pipeline.

The forward model depends on the location of the sources with respect to sensors, which changes between MEG runs as participants move. In this study, MEG recordings were pre-processed using MaxFilter, which realigns all session runs to a common head position. Therefore, we assumed the MEG forward model was the same for all session runs of a subject. We therefore computed the model only once then duplicated it across all runs for that subject.

#### Estimation of Noise Statistics From Empty-Room Recording (MEG)

Explicit inclusion of noise characteristics can benefit source estimation. For instance, minimum-norm estimators can include second-order sample statistics of sensor noise in the linear amplitude estimation of distributed sources. These statistics are summarized in an estimate of noise covariance between sensors, which takes the shape of a matrix. Brainstorm’s online documentation describes how this matrix can be estimated from EEG and MEG data. Here we estimated the MEG noise covariance from the empty-room measurements provided in the dataset. For EEG, we used the pre-stimulus baseline segments from all epochs.

For consistency, we preprocessed the empty-room recordings in an identical manner to the task data. We therefore applied the same notch filter before estimating the noise covariance matrix. We created a new subject called “sub-emptyroom” and linked all the empty-room data from folder derivatives/meg_derivatives/sub-emptyroom with menu “Review raw file” (preprocessed with MaxFilter/SSS). We then applied the same notch filters at 50, 100, 150, and 200 Hz. The estimation of the noise covariance for each session was obtained via a simple right-click over the filtered version of the empty-room data by selecting “Noise covariance > Compute from recordings.”

The noise environment outside the MEG and the state of the MEG sensors may change over time, it is therefore recommended to use empty-room data recorded just before or after the experimental recording itself, or at least from the same day (Gross et al., 2013). In this dataset, empty-room measurements were available for 8 different dates between April and December 2009. For each subject MEG session, we copied the noise covariance estimated from the closest empty-room recordings, as documented in the BIDS metadata (field “AssociatedEmptyRoom” associated to each acquisition run), i.e., for subject sub-01 we used empty-room ses-20090409 from April 9th, 2009.

#### Estimation of Noise Statistics From Pre-stimulus Baseline Data (EEG)

Empty-room recordings are not possible with EEG as electrodes need to be affixed to the scalp to pick up a signal. Noise covariance statistics also depend of the quality of each contact and therefore are specific to a given set of recordings. In the present study, our scientific hypothesis did not concern the possible role of pre-stimulus ongoing activity in task performance. Therefore, we considered these pre-stimulus data samples as noise and estimated their sample covariance across all trials. To do this we used time segments [-500, -0.9] ms concatenated across trials of the pre-processed, notch-filtered version of the data (Figure 8, left).

#### Weighted Minimum-Norm Estimation of Source Amplitudes

We used Brainstorm’s implementation of the weighted minimum-norm estimation (WMNE) of the amplitude of distributed sources, with default parameter settings suggested for regularization and source depth weighting. For technical details, please refer to (Baillet et al., 2001) and the online documentation^25^. WMNE has multiple options in selection of the source model (e.g., cortically constrained or volumetric), treatment of noise covariance and control of the regularizer that ensures a stable inverse solution. However, Brainstorm is configured with default values that take a conservative approach to source estimation and can safely be used with many if not most studies. Note that Brainstorm also features two other families of methods widely used in MEG/EEG: beamformers and equivalent dipole modeling but use of these is beyond the scope of the present manuscript. Imaging estimators such as WMNE provide well-studied solutions for subsequent statistical inference across participants and tend to be less user-dependent than equivalent dipole models. They are also computationally efficient as they can be implemented via the instantaneous linear combination of MEG/EEG sensor traces with a pre-computed kernel.

Weighted minimum-norm estimation maps can show bias with reduced source amplitude for radial source orientations in MEG and with increasing depth. This is partially addressed through the use of a depth-weighting (the Brainstorm default) but some bias remains. It is therefore common to standardize the WMNE current density estimates using information from the noise or data covariance, such as with dSPM (Dale et al., 1999) or sLORETA (Pascual-Marqui, 2002). These standardizations replace the current density estimate with a dimensionless statistic which can be used as the basis for hypothesis testing. Here we applied a simple standardization procedure based on the sample statistics of each source time series over the pre-stimulus baseline. In short, a *z*-score transformation was applied to each cortical source trace with respect to its pre-stimulus mean and standard deviation across time. We applied this transformation after trial averaging across runs, for each condition, and for each subject separately (see below).

Magnetoencephalography and EEG sensor data can be processed jointly to produce combined source estimates. Joint processing presents unique challenges because EEG and MEG use head models that exhibit differing sensitivities to modeling errors, which can in turn lead to inconsistencies between EEG and MEG with respect to the (common) source model. In practice joint processing is relatively rare (Baillet et al., 1999). However, these data are complementary, which means that joint processing can potentially yield insights that cannot be seen with either modality alone. For example, in the evoked responses in the data set used here, the first peak over the occipital areas is observed in MEG (90 ms) slightly before EEG (110 ms). This delay is too large to be caused by acquisition imprecisions. This indicates that we are not capturing the same brain processes with the two modalities, possibly because the orientation and type of activity in the underlying cortical sources is different. MEG and EEG have different sensitivities to source orientation and depth. Given the challenges of joint processing, our advice is to first look at the source reconstructions for the two modalities separately before trying to use any type of fusion technique. In the following, since our goal is to illustrate an end-to-end processing pipeline rather than comprehensively demonstrate all of Brainstorm’s features, we restrict our inverse results to MEG-only processing and do not present results either for EEG alone or joint MEG/EEG processing.

We used WMNE source mapping from “Compute sources [2018]” in Brainstorm, with the options: minimum-norm imaging, current density map, constrained normal to cortex, MEG MAG + MEG GRAD, and left all the advanced parameters values unchanged.

### Time-Frequency Decompositions

We computed the time-frequency decomposition of broadband MEG sensor data from each trial using Morlet wavelets. We then averaged the modulus of Morlet coefficients across trials for each condition and each run (Bertrand and Tallon-Baudry, 2000; Pantazis et al., 2005b). This operation was restricted to MEG magnetometers and EEG channels. The trials for the *Familiar* condition in run-01 were moved to the Process1 tab and the process “Frequency > Time-frequency (Morlet wavelets)”: with restriction to sensor types = MEG MAG, EEG was applied. Other parameters for the process were: not normalized, Frequency = log(6:20:60), which specifies that 20 frequency bins logarithmically spaced between 6 and 20 Hz are to be used, Measure = Power, Save average^26^. We repeated this procedure for the two other conditions (“Unfamiliar,” “Scrambled”).

**FIGURE 9:**
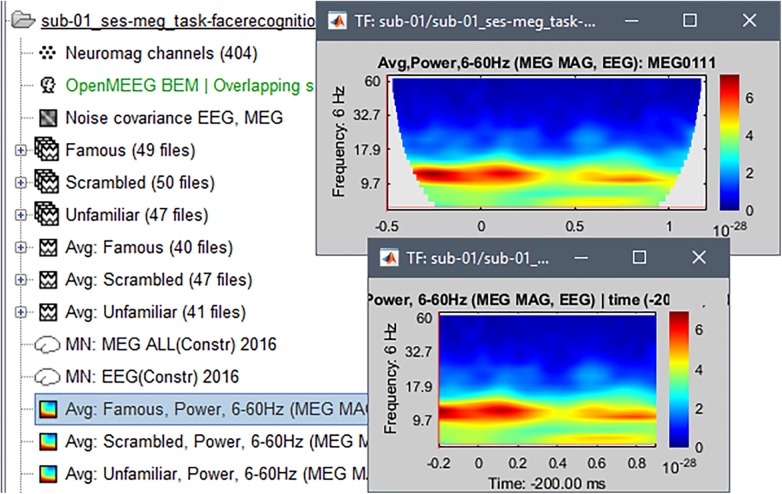
Time-frequency decomposition (Morlet wavelets) for channel EEG070, averaged over trials from the “Familiar” condition. The figure shows the outcome of the decomposition before **(top)** and after **(bottom)** removing signal edge effects. The TF plots for the other two conditions (“unfamiliar,” scrambled”) were visually indistinguishable from the one shown.

To avoid misinterpretation of power time-frequency decomposition values contaminated by signal edge effects, we selected the display option “Hide edge effects.” This latter revealed that time-frequency decompositions were reliable between -200 and +900 ms (Figure 9). In Process1, we selected these time-frequency results and ran process “Extract > Extract time,” with the option to overwrite input files. This latter step produced time-frequency decomposition data with interpretable values away from edge-effect contamination. Note that as shown in Figure 9 there is very little power present at frequencies above 15 Hz. Higher frequencies are typically of lower power, so that they are visible only after performing a frequency-dependent normalization as we describe below.

## Automation: Scripting and Reporting of Analysis Pipelines

Using the GUI is a convenient solution to explore options for data analysis on a subset of the data. We describe here how scripting can be used to reproduce efficiently an identical analysis pipeline on all runs and participants.

The Brainstorm pipeline editor automatically generates Matlab code from the “Generate.m script” menu item. The resulting script reproduces exactly the processing steps of the interactive GUI version of Brainstorm. “For” loops can be added to the script to batch the analysis across additional data runs, other participants or studies. We provide multiple online examples of such scripts, which can further help users assemble their own custom pipelines. Online recommendations and examples for editing Brainstorm scripts are available^27^.

One practical recommendation for Brainstorm scripting is to split data analysis pipelines in blocks. For instance, importing anatomical data and raw MEG/EEG files, frequency filtering, power spectrum estimation, artifact detection and cleaning do not require user interaction, and can all be processed at once from one single script. Interventions are required from users for reviewing raw files, marking/confirming bad channels and data segments, selecting SSP or ICA components, etc. Another batch script can then be generated for epoching and averaging data, producing source models and extracting measures of interest (e.g., time-frequency decompositions and connectivity metrics).

The outputs of executed scripts are consigned to a log, for verification of proper execution and debugging. Screen captures are added to this execution log via the “File > Save snapshot” menu item. Our recommendation is to add snapshots after the following key analytical steps: MEG/EEG-MRI registration, power spectrum density estimation, sensor topographies of selected SSP components, average event-related responses in all experimental conditions, sensor topography at the peak of the strongest primary visual response (≃110 ms).

Manual data processing steps are user dependent and therefore detrimental to the strict reproducibility of data analyses. However, fully automated pipelines have their own shortcomings when used blindly on rich, complex data. Manual data cleaning processes tend to be more specific than automated procedures, resulting in lesser amounts of data being rejected. For this reason, they remain the preferred approach for typical study designs in MEG/EEG research that involve relatively small cohorts of participants. We note, however, that big-data repositories are emerging in the field (e.g., OMEGA, MEG-HCP, and Cam-Can), which require more automated procedures, but put less emphasis on qualifying as much of the initial data volume as possible to subsequent analyses (Van Essen et al., 2012; Niso et al., 2016; Taylor et al., 2017).

Analysis pipelines that involve manual steps can be replicated, with additional care and documentation. For instance, the user-selected bad channels, bad segments and rejected spatial components need to be clearly documented. Following this idea, we provide a script that reports the rejected data segments for all 16 participants to the present study dataset^28^. The bad channels were identified manually; the bad segments were detected automatically, confirmed manually, then exported as text and copied at the end of this script. All process calls (via bst_process.m) used to produce the results shown here were generated with the pipeline editor, with few manual coding additions restricted to loops, bad channel identification and data file names. This script produced separate reports for each participant data; for illustration, we provide online the report produced by this script for subject sub-01^29^.

## Subject-Level Summary Statistics

Subject-level (i.e., across data runs) averages were obtained for event-related MEG responses, source maps and time-frequency decompositions, in each experimental condition separately^30^. As mentioned above, all data runs provided had been registered to a common head position with MaxFilter. This minimized the effect of different positions between runs in within-subject MEG sensor averages.

Unlike forward head models, source models were computed for each of the 6 runs separately before producing subject-level averages and other statistics. The reason for this is that source models consider effects of the SSP projectors applied and bad-channel selection that are typically specific to each data run.

### Condition-Specific Average Responses

For each experimental condition (*Familiar, Unfamiliar*, and *Scrambled*) and each subject, we derived event-related MEG signal averages. The subject-level average across all 6 runs was obtained by weighting each run-specific average by the number of good trials in each run. This approach reduces the influence of noisy runs, with a smaller number of good trials, on the subject-level average. For each data type (sensor data, source maps, and time-frequency decompositions), we used the Process1 panel after selecting all subject data from all runs. We then used process: “Average > Average files,” with the options: “By trial group (subject average), Weighted.” This produced three event-related averages (one per condition), for each subject, saved in their respective “intra-subject” folders in the Brainstorm database. Subject-level averages of MEG across runs were obtained separately.

### Event-Related Responses to Faces (Familiar and Unfamiliar)

Subject-level average responses to faces were generated for each data type (sensor data, source maps, and time-frequency decompositions) by averaging across the *Familiar* and *Unfamiliar* conditions, also using a weighted average approach. We used the Process2 tab to obtain these statistics for all subjects at once; this tab works similarly to Process1 except that it allows the user to specify two series of input files for processes that require two distinct data entries to operate. We selected all *Familiar* subject-level averages for the FilesA panel, and the *Unfamiliar* averages for the FilesB panel, following the same subject order for both file selections. We ran process “Other > Average A&B: Weighted” and added process “File > Set comment:” with option “WAvg: Avg: Faces,” to obtain the subject-level average data in response to the presentation of *Faces*.

Note that manual selection of many files from the database is difficult and prone to human errors. Brainstorm scripts can be used for the purpose of such database queries^31^.

### Contrasting Subject-Level Source Models

Special attention needs to be brought to contrasting source models. The reason is that they produce estimates of current amplitudes along elemental current dipoles, where the polarity reflects both the directionality of impressed current flow and possibly cross-talk contributions by more strongly active neighbor regions. Depending on whether the actual sign of dipole currents is of interest to the neuroscience question, two contrast measures between conditions A and B are commonly used in the field: the magnitude differences (|A|-|B|, agnostic to the current polarity), and amplitude differences [(A-B), which takes current polarity into consideration].

#### Magnitude Differences (|A|-|B| )

This measure highlights the difference in absolute current strengths between conditions, regardless of their polarity. The premise to this option is that polarity is of no interest to the scientific question and that source currents can be simply interpreted as brain *activation*. For instance, this option can be selected in the present case when testing whether responses to faces induce a greater activation in fusiform cortical regions than control images.

#### Amplitude Differences (A-B)

This measure should be used if the polarity of dipolar currents is relevant to the neuroscience question. For instance, this option can be selected when testing whether any oscillatory signal component can differentiate between responses to faces vs. control images. The interpretation of an A-B contrast with signed values is more ambiguous than with rectified measures, e.g., for identifying the experimental condition that produced the largest absolute brain response. For example, both (*A*,*B*) = (-10,-5) and (*A*,*B*) = (5,10) yield *A*-*B* = -5, we detect an effect but cannot identify which condition, from A and B, has produced the strongest response. On the other hand, dipoles with opposite directions are easy to detect with this difference, e.g., if (*A*,*B*) = (-10,10), |*A*-*B*| = 20, while |*A*| -|*B*| = 0. We provide online further discussion on these two alternative contrasts^32^.

To test when and where the amplitude of brain activity differed between the presentation of faces vs. scrambled images, we used the Process2 tab to obtain the difference between subject-level average source data for the MEG data. We dropped all the Faces subject averages in FilesA, and all the Scrambled averages in FilesB. We selected the process “Difference > Difference A-B: Do not use absolute values” and added the process “File > Set comment: “Faces – Scrambled|MEG””. We repeated the same procedure to obtain the difference between the *Familiar* and *Unfamiliar* conditions for each subject.

### Low-Pass Filtering

**FIGURE 10:**
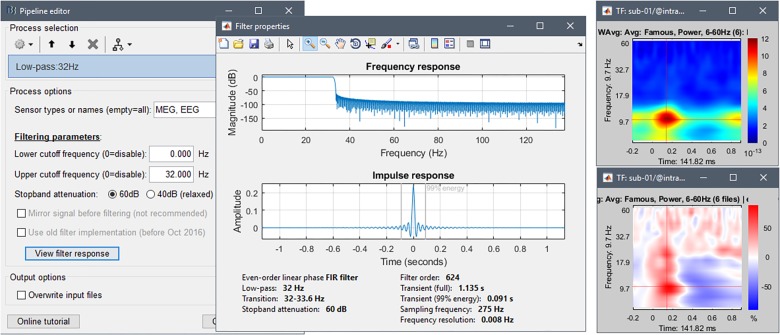
**(Left)** Options user interface for process “Band-pass filter”. **(Right)** Time-frequency decomposition of channel EEG070 for the Familiar condition, before **(top)** and after **(bottom)** baseline normalization.

To reproduce the approach of Wakeman and Henson (2015) we low-pass filtered the trial-averaged sensor data and source maps below 32 Hz. To evaluate the duration of filter transients with respect to epoch duration, we visualized the filter impulse response from the GUI option of Brainstorm’s “Band-pass filter” process (Figure 10, and online resource^33^). The procedure indicates the full duration of the filter transients (here 1135 ms) and the duration containing 99% of the energy of the filter response (here 91 ms). We decided to crop 300 ms at the beginning and end of each epoch (original epoch: -500 to 1200 ms), which does not concern the signal latencies of interest to the present study. As mentioned above, ideally, decisions concerning temporal filters derive from the hypotheses to be tested with the data. Hence filtering may be advantageously performed on the ongoing data before epoching, which produces edge effects at the beginning and end of the recording, not of each epoch.

We used the Process1 tab to select all the intra-subject folders from all participants. These folders contain the condition and subject specific averages. For each data type (sensor data and source maps), we ran the process “Pre-process > Band-pass filter” with options passband: 0–32 Hz, data types: MEG, EEG, 60 dB attenuation, and file Overwrite, followed by “Extract > Extract time” with option [-200, 900] ms - this latter to remove filter edge effects, as explained above.

### Inter-Individual and Cross-Frequency Standardization

In the same experimental conditions, and with similar behavioral performances, the intensity of neural currents vary between individuals because of anatomical and physiological differences that are of no primary interest to the study. Also, for MEG source maps, estimated current strengths are weaker where signal sensitivity drops, for instance for radial or deeper sources. If time-frequency decompositions are to be analyzed, cross-frequency standardization of magnitude changes is also required, to compensate for the typical 1/*f* decrease in electrophysiology signal amplitude.

Standardization procedures with respect to baseline levels lessen the influence of these factors of no interest. We therefore applied a *Z*-transformation on source time series with respect to pre-stimulus baseline activity. We selected all subject-level source map averages in Process1 and ran the process “Standardize > Baseline normalization” with options Baseline = [-200,-5] ms, *Z*-score, Overwrite. We also standardized the magnitude of time-frequency decompositions for each subject and condition with event-related synchronization/desynchronization (ERS/ERD) scaling. This procedure centers and normalizes the modulus of wavelet coefficients for each frequency bin, with respect to their sample mean over baseline, as shown in Figure 10.

We distribute a Brainstorm database that contains the outputs of all analyses described so far (Frontiers2018Group.zip, 14 Gb, available from Brainstorm’s download page^34^). These outputs can be reproduced by running the Matlab scripts tutorial_frontiers2018_single.m^35^ and tutorial_frontiers2018_copy.m^36^, distributed with Brainstorm. The entire multisubject data volume can readily be imported into Brainstorm via the “File > Load protocol > Load from zip file” menu selection. For bandwidth considerations, the distributed files were downsampled to 275 Hz.

## Group Analyses

We derived inferential statistics for two contrasts of interest: *Faces* vs. *Scrambled* and *Familiar* vs. *Unfamiliar* faces. According to Wakeman and Henson (2015), we expected to observe stronger bilateral event-related responses in occipital visual cortex (V1) and occipital face and fusiform face areas (OFA and FFA) within 170 ms in response to stimuli containing faces vs. the scrambled data; we also anticipated augmented activation over the right superior temporal sulcus region (STS) in the *Familiar* vs. *Unfamiliar* condition around a 250-ms latency. These procedures are reproducible automatically using the function tutorial_frontiers2018_group.m^37^.

### Group-Level Sensor Data

#### Grand Averages Across Participants

We produced grand arithmetic averages of sensor data across participants for the *Faces, Scrambled, Familiar*, and *Unfamiliar* conditions. We selected in Process1 all the MEG/EEG subject-level average files from the participant-specific “Intra-subject” folders in the Brainstorm database. We then ran the process “Average > Average files” with options “By trial group (grand average), Not weighted.”

**FIGURE 11:**
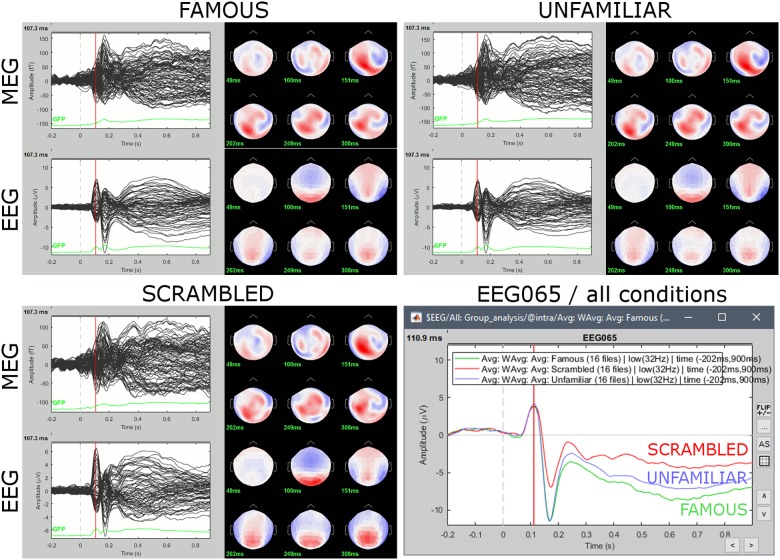
Grand averages for the three experimental conditions for both EEG (μV) and MEG (fT), and for a single parieto-occipital electrode (EEG065). Sensor topography maps show grand-average (group-level) MEG and EEG data at selected latencies: 50, 100, 150, 200, 250, and 300 ms. Bottom-right panel emphasizes signal differences at electrode EEG065 between conditions. Subsequent group-level inferential statistics will test for significant differences between experimental conditions.

As anticipated, we observed in EEG a greater negative component around 170 ms for *Faces* vs. *Scrambled*, and sustained signal differences after 250 ms between the *Familiar* and *Unfamiliar* conditions (Figure 11).

We computed the differences of grand averages between conditions of interest using the Process2 tab for selecting the grand averages for *Faces* (FilesA) and *Scrambled* (FilesB). We then ran the process “Other > Difference A–B”. The same procedure was repeated for *Familiar* vs. *Unfamiliar*.

We observed signal differences in both contrasts: after 160 ms for *Faces* vs. *Scrambled*, and after 200 ms for *Familiar* vs. *Unfamiliar*. We then performed statistical inference on the significance of these differences, using parametric and non-parametric approaches. We chose a type-I error rate of *α* = 5% with correction from multiple comparisons by adjustment of the false discovery rate (FDR).

#### Non-parametric Statistical Inference

**FIGURE 12:**
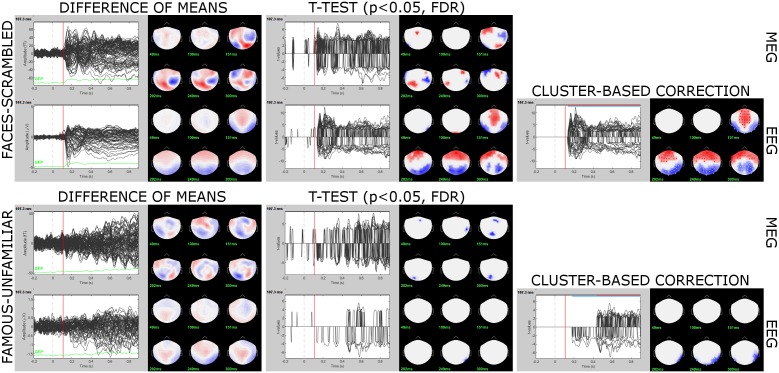
Sensor-level contrasts Faces vs. Scrambled **(top)** and Familiar vs. Unfamiliar **(bottom)**. The output of non-parametric *t*-tests are *t*-value traces that are set to 0 at every channel and time point where *p* > 0.05 (FDR-corrected). The non-zero values from the cluster-based permutation test results revealed a difference in spatiotemporal adjacency of the sensor data between the two tested conditions.

Brainstorm features a toolkit for parametric and non-parametric inferential statistical testing^38^ . Here we present the application of a non-parametric procedure using permutation testing. Although more computationally demanding, it is a more robust approach than parametric tests (Pantazis et al., 2005a). We selected in Process2 the 16 subject averages for *Faces* (FilesA) and *Scrambled* (FilesB) and ran process “Test > Permutation test” with options “Paired: *t*-test and “1000 randomizations.” We repeated the procedure for *Familiar* vs. *Unfamiliar* (Figure 12).

For completeness of the illustration of possible workflows produced with Brainstorm, we also applied cluster correction to sensor data contrasts (Maris and Oostenveld, 2007). We used Brainstorm’s capacity to execute code from the FieldTrip toolbox (Oostenveld et al., 2011). Brainstorm structures are converted dynamically to FieldTrip structures, the FieldTrip code is executed, and the returned structures are converted into Brainstorm database entries.

### Group-Level Source Maps

We now describe the procedure to produce inferential statistics on source maps for the *Faces* vs. *Scrambled* contrast (same approach would apply to *Familiar* vs. *Unfamiliar*).

#### Anatomical Standardization Between Participants

**FIGURE 13:**
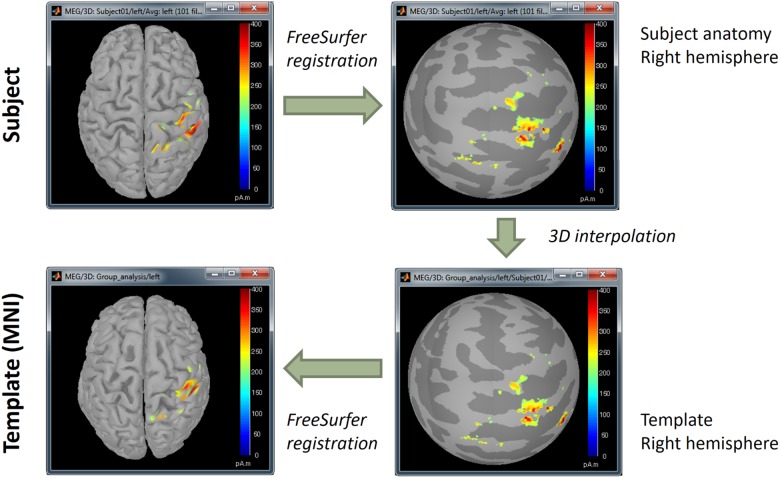
Surface-based coregistration procedure by alignment of curvature maps in spherical topology.

We mapped all individual source maps to the MNI/ICBM152 brain template (Fonov et al., 2009)^39^, available in the “default anatomy” folder of the Brainstorm protocol. Brainstorm uses the surface-based registration approach from FreeSurfer, based on a spherical representation of the cortex topology. Note that this feature is available in Brainstorm when the imported MRI data is processed with FreeSurfer^40^ (Figure 13). A similar approach is available for data processed with BrainSuite.

We aligned the individual source maps consisting of the absolute values of current amplitudes to assess differential effects in brain activation between experimental conditions (see above).

We selected in Process1 all 16 intra-subject folders and ran process “Pre-process > Absolute values” followed by process “Sources > Project on default anatomy.” The projected source maps were automatically regrouped in a single Brainstorm folder labeled “Group analysis/Intra-subject.” Note that new folders can be created by users for customized data organization, e.g., with one folder per experimental condition.

#### Spatial Smoothing

To reduce noise and ameliorate the impact of individual variations in functional specialization relative to cortical anatomy, we applied spatial smoothing of the resulting source maps to further reduce inter-individual variability across the group. Smoothing is performed using a Gaussian kernel scaled to the size of edges on the cortical mesh. We selected in Process1 all the projected source maps and ran process “Sources > Spatial smoothing” with options “FWHM = 3 mm” (FWHM = Full Width Half maximum) and “Overwrite”. This process relies on the function ‘SurfStatSmooth,’ implemented in SurfStat (Worsley et al., 2009).

#### Contrasting Group-Level Source Maps

**FIGURE 14:**
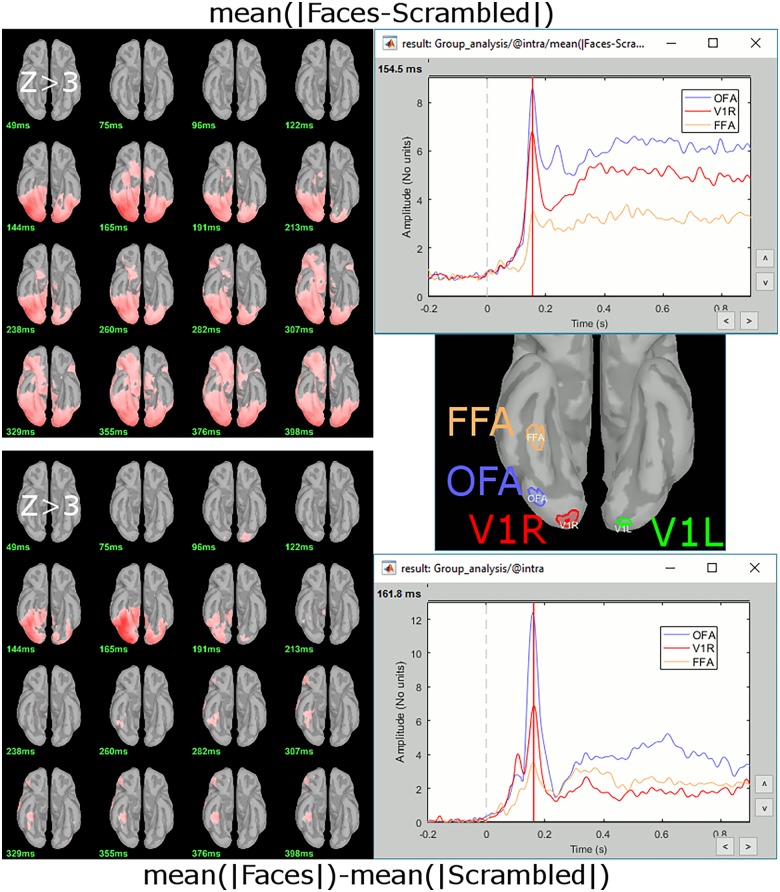
Various measures associated to the contrast (Faces-Scrambled) for MEG sources. **(Top)** Average of within-subject differences, normalized source maps at various latencies from 50 to 400 ms for *Z* > 3, and time series extracted from three ROIs of the ventral visual stream. **(Bottom)** Difference of grand averages, source maps and ROI time series.

Contrast maps of *z*-scored cortical sources in the Faces vs. Scrambled face conditions showed enhanced responses for Faces in occipital and ventral stream regions, including in the fusiform face area (FFA; Figure 14). The analysis used both the amplitude difference approach (Figure 14, top) and the magnitude difference approach (Figure 14, bottom). Stronger activity for Faces in the most posterior regions was initiated before FFA, with contrast activations in all ROIs peaking around 155 ms.

## Conclusion

Brainstorm provides interactive and user-friendly tools to design automated and standardized processing pipelines, with an emphasis on quality control and verification of data integrity. The application features convenient tools to perform visual inspection of the outcome of all steps of a data analysis pipeline via execution reports. The analyses presented here are entirely reproducible via the following procedure:

(1) Download the data in tutorial_dir (170 Gb): https://openneuro.org/datasets/ds000117.
(2) Note that getting the data from a web browser as a single zip file did not work well at time of submission, another more reliable solution using the Amazon AWS CLI software is described on the online tutorials^41^.
(3) In this analysis, we used only the “derivatives” folder (85 Gb), all the other folders can be safely deleted if disk space is an issue.
(4) The execution of the pipeline scripts requires a Matlab license: you may use any Matlab version from 2008b to 2018b, except for 2018a because of a bug in Matlab’s svd function.
(5) Download and install Brainstorm^42^. In general, we recommend getting the most up-to-date version available from the Brainstorm website, however, for the strict reproducibility of the results presented in this article, we uploaded a development snapshot from November 11th, 2018, on the Zenodo website^43^.
(6) For cluster-based statistics, we used functions from the FieldTrip toolbox. Download FieldTrip^44^ and add it to the Matlab path. If you are using the Brainstorm version from Zenodo, the repository also includes the FieldTrip version we used for the computation (December 17, 2017).
(7) Start Brainstorm, set the database folder as instructed in the installation instructions.
(8) In the Brainstorm window, select menu File > Edit preferences: Edit the paths to the temporary folder (if you have limited space in your user’s home folder) and to the FieldTrip toolbox.
(9) Close Brainstorm.
(10) Create an empty folder to store the execution reports, outside of any of the Brainstorm folders (reports_dir).
(11) The total size of the Brainstorm database after processing will be around 130 Gb, make sure enough space is available on the hard drive.
(12) In your Matlab command window, type: tutorial_frontiers2018(tutorial_dir, reports_dir). This will run all the scripts mentioned in this article: tutorial_frontiers2018_single.m, tutorial_frontiers2018_copy.m, tutorial_frontiers2018_group.m. All these scripts are located in the folder brainstorm3/toolbox/script, which is created and added to the user Matlab path after starting Brainstorm. Execution time is typically between 10 and 30 h, depending on hardware. For detailed execution times on the reader’s system, please refer to the reports saved in reports_dir; examples for the execution on a Dell XPS 2016 laptop are available from the online tutorials^45^.
(13) For keeping the execution time reasonable, some processes described in this article that have no or very little impact on the final results have been commented out, identified with the label “SHORT VERSION” in the scripts. The skipped steps are the following: import of the FreeSurfer ASEG atlas, notch filtering, EEG BEM forward model with OpenMEEG, individual source snapshots in the execution reports, time-frequency analysis. To enable a step, delete the comment marker “%” at the beginning of all the lines in the code section. Additionally, the recordings and source results have been downsampled to 275 Hz before group analysis.

## Author Contributions

JM, DP, RL, and SB contributed the basic methods. RL, JM, DP, SB, and FT designed the software features. FT developed the software and documentation. EB contributed data preprocessing and quality assurance. GN deployed the efforts for data standardization. FT, SB, and RL wrote the manuscript. All authors contributed to manuscript revision.

## Conflict of Interest Statement

The authors declare that the research was conducted in the absence of any commercial or financial relationships that could be construed as a potential conflict of interest.

## Footnotes

1 ftp://ftp.mrc-cbu.cam.ac.uk/personal/rik.henson/wakemandg_hensonrn/Publications/SPM12_manual_chapter.pdf

2 http://bids.neuroimaging.io

3 See online tutorials MEG visual: single subject (Elekta/BIDS) and MEG visual: group study (Elekta/BIDS).

4 Introduction in 28 parts for new Brainstorm users: http://neuroimage.usc.edu/brainstorm/Tutorials#Get_started

5 http://surfer.nmr.mgh.harvard.edu

6 Installation instructions: http://neuroimage.usc.edu/brainstorm/Installation.

7 See online tutorial MEG resting state and OMEGA database.

8 See online tutorial Using FreeSurfer for more information and software capabilities.

9 See online tutorial MEG-MRI coregistration, and Section “Registration With Structural MRI.”

10 derivatives/meg-derivatives/sub-01/ses-meg/meg/sub-01_ses-meg_task-facerecognition_run-01_proc-sss_meg.fif

11 See online tutorial Review continuous recordings.

12 See online tutorials Event markers and Select files and run processes.

13 See online tutorial: Power spectrum and frequency filters.

14 See online tutorial Power spectrum and frequency filters.

15 See online tutorial Bad channels.

16 See online tutorial Montage editor for alternative approaches.

17 See online tutorial Artifact detection.

18 See online tutorial Artifact cleaning with SSP.

19 See online tutorial Import epochs.

20 See online tutorials Average response, Visual exploration, Colormaps, and Clusters of sensors.

21 See online tutorials Head modeling, Noise covariance, Source estimation, Dipole scanning, and Dipole fitting.

22 See online tutorial Deep cerebral structures.

23 See online tutorials “Volume source estimation” (https://neuroimage.usc.edu/brainstorm/Tutorials/TutVolSource) and “Warping the anatomy templates” (https://neuroimage.usc.edu/brainstorm/Tutorials/TutWarping)

24 See online tutorial Realistic head model: BEM with OpenMEEG.

25 See online tutorial Source estimation.

26 See online tutorial Time-frequency.

27 See online tutorial Scripting.

28 https://github.com/brainstorm-tools/brainstorm3/blob/master/toolbox/script/tutorial_frontiers2018_single.m

29 https://neuroimage.usc.edu/bst/examples/report_Frontiers2018Single_sub-01.html

30 See online tutorial Workflows.

31 Processes “File > Select files”, documented in online tutorial Scripting.

32 See online tutorials Difference and Workflows.

33 See section View filter response in tutorial “Power spectrum and frequency filters.”

34 http://neuroimage.usc.edu/bst/download.php

35 https://github.com/brainstorm-tools/brainstorm3/blob/master/toolbox/script/tutorial_frontiers2018_single.m

36 https://github.com/brainstorm-tools/brainstorm3/blob/master/toolbox/script/tutorial_frontiers2018_copy.m

37 https://github.com/brainstorm-tools/brainstorm3/blob/master/toolbox/script/tutorial_frontiers2018_group.m

38 See online tutorial Statistics.

39 http://www.bic.mni.mcgill.ca/ServicesAtlases/ICBM152NLin2009

40 See online tutorial Group analysis: Subject coregistration.

41 https://neuroimage.usc.edu/brainstorm/Tutorials/VisualSingle#Download_and_installation

42 http://neuroimage.usc.edu/brainstorm/Installation

43 https://zenodo.org/record/1479794

44 http://www.fieldtriptoolbox.org/download

45 https://neuroimage.usc.edu/bst/examples/report_Frontiers2018Single_sub-01.html, https://neuroimage.usc.edu/bst/examples/report_TutorialGroup_2snapshots.html, https://neuroimage.usc.edu/bst/examples/report_TutorialGroup_3meeg.html, https://neuroimage.usc.edu/bst/examples/report_TutorialGroup_4sources.html
